# Impact of small for gestational age on length of hospital stay and hospitalization costs in very preterm infants: a single-center retrospective cohort study

**DOI:** 10.1186/s12887-026-07284-6

**Published:** 2026-07-15

**Authors:** Zhihong Wang, Xiaoyun Chen, Licong Lin

**Affiliations:** 1 Department of Neonatology, Zhangzhou Affiliated Hospital of Fujian Medical University, Zhangzhou, Fujian 363000 China; 2 Department of Anesthesiology, Zhangzhou Affiliated Hospital of Fujian Medical University, Zhangzhou, Fujian 363000 China

**Keywords:** Small for gestational age, Very preterm infant, Length of stay, Hospitalization costs, Retrospective cohort study

## Abstract

**Background:**

Small for gestational age (SGA) is common among very preterm infants, but its independent impact on healthcare resource utilization remains controversial. This study aimed to evaluate the independent effect of SGA on length of hospital stay (LOS) and hospitalization costs in very preterm infants.

**Methods:**

This single-center retrospective cohort study included preterm infants born at < 32 weeks of gestation and admitted to the NICU of Zhangzhou Affiliated Hospital of Fujian Medical University between January 2024 and February 2026. Infants were classified as SGA (birth weight < 10th percentile for gestational age and sex according to the Fenton 2013 growth chart) or non-SGA. The primary outcome was LOS; secondary outcomes included hospitalization costs, duration of respiratory support, and discharge anthropometrics. Multivariable generalized linear models with Gamma distribution and log link function were used to assess the independent association between SGA status and outcomes, adjusting for gestational age, mode of delivery, invasive mechanical ventilation duration, and bronchopulmonary dysplasia.

**Results:**

A total of 270 very preterm infants were included, of whom 17 (6.3%) were classified as SGA. The SGA group had significantly longer LOS (median: 68.0 vs. 48.0 days, *P* = 0.022) and higher hospitalization costs (median: 96,219 vs. 71,361 RMB, *P* = 0.044) compared with the non-SGA group. After adjusting for gestational age, cesarean delivery, invasive mechanical ventilation duration, and bronchopulmonary dysplasia, SGA remained independently associated with prolonged LOS (approximately 40% longer; Exp(B) = 1.401, 95% CI: 1.239–1.582, *P* < 0.001) and increased hospitalization costs (approximately 34% higher; Exp(B) = 1.339, 95% CI: 1.112–1.614, *P* = 0.002).

**Conclusions:**

SGA is an independent risk factor for prolonged LOS (approximately 40% increase) and increased hospitalization costs (approximately 34% increase) in very preterm infants, after adjusting for illness severity. These findings provide quantitative evidence for healthcare resource planning and targeted management of this high-risk population.

## Background

With advances in perinatal medicine and neonatal intensive care, the survival rate of very preterm infants (gestational age < 32 weeks) has improved substantially [1]. However, these infants require prolonged hospitalization and incur substantial healthcare costs, imposing a heavy burden on families and healthcare systems [2–4]. Among this high-risk population, small for gestational age (SGA) accounts for a considerable proportion, with a reported incidence of approximately 10%–20% [5–7].

SGA is defined as birth weight below the 10th percentile for gestational age and sex, resulting from intrauterine growth restriction and other factors [8]. In China, the incidence and associated problems of SGA have attracted growing attention [9]. Existing evidence on whether SGA increases the risk of adverse outcomes in preterm infants remains conflicting. Some studies have reported that SGA is associated with an increased risk of bronchopulmonary dysplasia, necrotizing enterocolitis, and mortality [10–12], whereas others have suggested that chronic intrauterine stress may accelerate lung maturation, conferring a potential protective effect on respiratory outcomes [13]. A recent multicenter study from Shenzhen, China reported substantial variations in LOS among surviving preterm infants in NICUs [14]; however, the specific contribution of SGA status to LOS and hospitalization costs was not the primary focus of that analysis. Data on the combined impact of SGA on both LOS and hospitalization costs—two direct indicators of healthcare resource utilization—remain limited.

Therefore, this study aimed to evaluate the independent effect of SGA on LOS and hospitalization costs in a single-center cohort of very preterm infants, providing evidence for clinical resource planning and targeted management of SGA infants.

## Methods

### Study design and population

This was a single-center retrospective cohort study reported in accordance with the Strengthening the Reporting of Observational Studies in Epidemiology (STROBE) statement [15]. All preterm infants born at < 32 completed weeks of gestation and admitted to the neonatal intensive care unit (NICU) of Zhangzhou Affiliated Hospital of Fujian Medical University between January 1, 2024, and February 28, 2026, were consecutively screened for eligibility.

Inclusion criteria were: (1) gestational age < 32 weeks; (2) born alive and admitted to the NICU; and (3) complete medical records available. Exclusion criteria were: (1) major congenital anomalies; (2) LOS < 2 days (palliative care or early death); and (3) missing data on SGA status, gestational age, or key outcome variables.

Data were retrospectively extracted from the hospital’s electronic medical record system. Data collection was completed by February 28, 2026. This study was approved by the Medical Ethics Committee of Zhangzhou Affiliated Hospital of Fujian Medical University in April 2026 (Approval No.: 2026LWB166), after completion of data extraction. The requirement for informed consent was waived due to the retrospective nature of the study.

### Data collection

Data were retrospectively extracted from the hospital’s electronic medical record system. The following variables were collected: maternal and obstetric variables (gestational age, mode of delivery, antenatal corticosteroid administration, gestational diabetes mellitus, chorioamnionitis, assisted reproduction); neonatal variables (sex, birth weight, SGA status, surfactant use); and outcome variables (duration of invasive mechanical ventilation, duration of non-invasive ventilation, total duration of respiratory support and oxygen therapy, LOS, total hospitalization costs, discharge weight, discharge head circumference).

### Definitions

Small for gestational age (SGA) was defined as birth weight below the 10th percentile for gestational age and sex according to the Fenton 2013 growth chart, which is the most widely used international growth reference for preterm infants and facilitates cross-study comparisons [8]. Gestational age was determined based on first-trimester ultrasound or the last menstrual period and expressed in completed weeks and days (converted to decimal weeks for analysis: weeks + days/7). Complete antenatal corticosteroid course was defined as administration of two doses of betamethasone 24 h apart or four doses of dexamethasone 12 h apart, completed at least 24 h before delivery [16]. Length of hospital stay was defined as the number of days from birth to discharge. Total hospitalization costs were defined as the total medical expenses incurred during the entire NICU admission, expressed in Chinese Yuan (RMB).

### Outcomes

The primary outcome was LOS (days). Secondary outcomes included: (1) total hospitalization costs (RMB); (2) total duration of respiratory support (days); (3) duration of invasive mechanical ventilation (days); (4) duration of non-invasive ventilation (days); (5) discharge weight (g); and (6) discharge head circumference (cm).

### Statistical analysis

Continuous variables were tested for normality using the Shapiro-Wilk test. As most outcome variables were non-normally distributed, comparisons between the SGA and non-SGA groups were performed using the Mann-Whitney U test for continuous variables and the Chi-square test or Fisher’s exact test for categorical variables, as appropriate. For normally distributed continuous variables, Student’s t-test was used.

Generalized linear models (GLM) with Gamma distribution and log link function were used to assess the independent association between SGA status and the primary outcome (LOS) and the key secondary outcome (hospitalization costs). The Gamma distribution with log link was selected because LOS and cost data are strictly positive, right-skewed continuous variables for which this approach is methodologically appropriate [17, 18]. Model coefficients (B) were exponentiated to obtain Exp(B), representing the proportional change in the outcome associated with each predictor.

Covariates included in the models were gestational age (continuous, days), mode of delivery (cesarean vs. vaginal), invasive mechanical ventilation (IMV) duration (continuous, days), and bronchopulmonary dysplasia (BPD, yes/no). These covariates were selected based on clinical relevance as established drivers of healthcare resource utilization in very preterm infants. Birth weight was not included due to its high collinearity with SGA status. Data on necrotizing enterocolitis (NEC) and sepsis were not systematically collected and therefore could not be included.

All statistical analyses were performed using SPSS version 23.0 (IBM Corp., Armonk, NY, USA). A two-sided P value < 0.05 was considered statistically significant.

## Results

### Study population

A total of 270 very preterm infants were included in the final analysis, of whom 17 (6.3%) were classified as SGA and 253 (93.7%) as non-SGA.

### Baseline characteristics

The baseline characteristics of the two groups are summarized in Table 1. Infants in the SGA group had significantly higher gestational age compared with the non-SGA group (30.57 ± 2.28 vs. 29.49 ± 1.81 weeks, *P* = 0.020) and, by definition, significantly lower birth weight (925.29 ± 224.70 vs. 1309.82 ± 321.43 g, *P* < 0.001). The rate of cesarean delivery was significantly higher in the SGA group (88.2% vs. 57.7%, *P* = 0.013). There were no statistically significant differences between the two groups with respect to sex distribution, complete antenatal corticosteroid course, gestational diabetes mellitus, chorioamnionitis, assisted reproduction, or surfactant use (all *P* > 0.05).

**Table 1:** Baseline characteristics of SGA and non-SGA very preterm infants

Characteristic	SGA (*n* = 17)	Non-SGA (*n* = 253)	*P* value
Gestational age (weeks), mean ± SD	30.57 ± 2.28	29.49 ± 1.81	0.020
Birth weight (g), mean ± SD	925.29 ± 224.70	1309.82 ± 321.43	< 0.001
Male sex, n (%)	12 (70.6)	143 (56.5)	0.316
Cesarean delivery, n (%)	15 (88.2)	146 (57.7)	0.013
Complete antenatal steroids, n (%)	11 (64.7)	121 (47.8)	0.178
Gestational diabetes mellitus, n (%)	9 (52.9)	81 (32.0)	0.076
Chorioamnionitis, n (%)	12 (70.6)	171 (67.6)	0.798
Assisted reproduction, n (%)	2 (11.8)	30 (11.9)	1.000
Surfactant use, n (%)	13 (76.5)	217 (85.8)	0.292

P values from Student’s t-test for continuous variables and Chi-square test or Fisher’s exact test for categorical variables

### Univariate analysis of outcomes

The univariate analysis of in-hospital outcomes is presented in Table 2. The SGA group had significantly longer LOS (median: 68.0 days, IQR: 46.5–97.0) than the non-SGA group (median: 48.0 days, IQR: 36.0–67.0; mean rank: 177.35 vs. 132.69, *P* = 0.022) and significantly higher hospitalization costs (median: 96,218.60 RMB, IQR: 65,823.80–150,393.44) than the non-SGA group (median: 71,361.15 RMB, IQR: 47,277.15–102,416.08; mean rank: 172.41 vs. 133.02, *P* = 0.044).Total duration of respiratory support was numerically higher in the SGA group (mean rank: 162.97 vs. 133.65), but this difference did not reach statistical significance (*P* = 0.134). No statistically significant differences were observed between the two groups in duration of invasive mechanical ventilation, non-invasive ventilation, discharge weight, or discharge head circumference (all *P* > 0.05).

**Table 2:** Univariate analysis of in-hospital outcomes

Outcome	SGA Median (IQR)	Non-SGA Median (IQR)	SGA Mean Rank	Non-SGA Mean Rank	*P* value
Invasive ventilation (days)	—	—	127.21	136.06	0.624
Non-invasive ventilation (days)	—	—	141.06	135.13	0.762
Total respiratory support (days)	—	—	162.97	133.65	0.134
Length of hospital stay (days)	68.0 (46.5–97.0)	48.0 (36.0–67.0)	177.35	132.69	0.022
Hospitalization costs (RMB)	96,218.60 (65,823.80–150,393.44)	71,361.15 (47,277.15–102,416.08)	172.41	133.02	0.044
Discharge weight (g)	—	—	123.09	135.27	0.530
Discharge head circumference (cm)	—	—	148.97	133.52	0.422

P values from Mann-Whitney U test. Mean ranks are reported due to non-normal distribution of outcome variables. For LOS and hospitalization costs, medians and interquartile ranges (IQR) are also provided as requested. — indicates that medians were not specifically requested for these secondary outcomes

### Multivariable regression analysis

In the generalized linear models with Gamma distribution and log link function, after adjusting for gestational age, cesarean delivery, IMV duration, and BPD, SGA remained independently associated with prolonged LOS (Exp(B) = 1.401, 95% CI: 1.239–1.582, *P* < 0.001) and increased hospitalization costs (Exp(B) = 1.339, 95% CI: 1.112–1.614, *P* = 0.002) (Table 3). Gestational age was associated with decreased LOS (Exp(B) = 0.859 per day, 95% CI: 0.842–0.877, *P* < 0.001) and costs (Exp(B) = 0.858 per day, 95% CI: 0.832–0.885, *P* < 0.001). Cesarean delivery was significantly associated with slightly shorter LOS (Exp(B) = 0.925, *P* = 0.011) but not with costs (*P* = 0.892). IMV duration and BPD were both significantly associated with increased LOS and costs.

**Table 3:** Generalized linear models (Gamma distribution with log link) for LOS and hospitalization costs

Outcome / Variable	B	Exp(B)	95% CI for Exp(B)	Wald χ²	*P* value
Length of hospital stay (days)					
Gestational age (per day)	−0.152	0.859	0.842–0.877	213.63	< 0.001
Cesarean delivery (vs. vaginal)	−0.078	0.925	0.871–0.982	6.46	0.011
SGA (vs. non-SGA)	0.337	1.401	1.239–1.582	29.08	< 0.001
IMV duration (per day)	0.003	1.003	1.000–1.005	5.35	0.021
BPD (vs. no BPD)	0.246	1.279	1.190–1.375	45.23	< 0.001
Hospitalization costs (RMB)					
Gestational age (per day)	−0.153	0.858	0.832–0.885	94.78	< 0.001
Cesarean delivery (vs. vaginal)	−0.006	0.994	0.908–1.087	0.02	0.892
SGA (vs. non-SGA)	0.292	1.339	1.112–1.614	9.47	0.002
IMV duration (per day)	0.005	1.005	1.001–1.009	5.67	0.017
BPD (vs. no BPD)	0.294	1.342	1.204–1.496	28.41	< 0.001

Exp(B) interpretation: Values > 1 indicate proportionally longer LOS/higher costs; values < 1 indicate proportionally shorter LOS/lower costs

## Discussion

In this single-center retrospective cohort study of 270 very preterm infants, SGA was identified as an independent risk factor for both prolonged LOS and increased hospitalization costs. After adjusting for gestational age, mode of delivery, IMV duration, and BPD, SGA infants’ LOS was approximately 40% longer and their costs approximately 34% higher compared with non-SGA infants.

The finding that SGA prolongs LOS may not be fully explained by lower birth weight requiring more time to reach discharge weight. We acknowledge that achieving a target discharge weight is an important contributing factor to prolonged LOS in SGA infants. However, after adjusting for gestational age—the strongest driver of growth and maturation—as well as IMV duration and BPD, SGA status still demonstrated a substantial independent effect (Exp(B) = 1.401, *P* < 0.001), suggesting that SGA infants may face additional intrinsic delays in achieving discharge readiness. Achieving discharge readiness involves the maturation of multiple physiological competencies, including full oral feeding, thermal stability, and respiratory stability [19]. Among these, the establishment of full oral feeding is often the final barrier to discharge. A comprehensive review highlighted that the maturation of suck-swallow-breathe coordination is a complex neurodevelopmental process, and delays in this process frequently prolong hospitalization in preterm infants [20]. This has been further corroborated by a randomized controlled trial demonstrating that swallowing exercises and non-nutritive sucking significantly improve oral feeding readiness in preterm infants [21], confirming the critical role of feeding maturation in discharge timing from an interventional perspective. SGA infants are predisposed to feeding intolerance due to relative intestinal hypoperfusion from chronic intrauterine hypoxia [22]; they also have reduced brown fat reserves and a larger surface-area-to-body-mass ratio, delaying the achievement of independent thermoregulation. These compounding challenges may contribute to prolonged hospitalization independently of birth weight itself [23]. Carducci and Bhutta also noted in a comprehensive review that the care of growth-restricted newborns requires simultaneous attention to feeding, thermoregulation, infection prevention, and developmental support—delay in any single dimension can postpone discharge [24]. Furthermore, altered neurobehavioral maturation in SGA infants may further delay the establishment of full oral feeding [25].

A recent multicenter study from Shenzhen reported substantial variations in LOS among surviving preterm infants in NICUs [14], but did not specifically isolate the effect of SGA. Our study extends this evidence by quantifying the independent contribution of SGA to both LOS and hospitalization costs. Notably, recent evidence has linked neonatal morbidities with postnatal growth faltering in preterm neonates [26], and SGA has been identified as an independent risk factor for post-discharge growth retardation among very-low-birth-weight infants in a nationwide Taiwanese registry study [27], suggesting that SGA infants may face ongoing growth challenges even after achieving comparable discharge anthropometrics.

From a health economics perspective, preterm birth itself is a well-established driver of high healthcare expenditures [2, 3]. Globally, the majority of NICU resources are devoted to the care of preterm infants, whose hospitalization costs account for over 50% of total NICU expenditures [28]. Research from the International Network for Evaluating Outcomes (iNeo) of neonates has repeatedly confirmed that improving outcomes and reducing long-term healthcare costs for very preterm infants is a global priority [29]. Our study demonstrates that, even within the very preterm population, SGA status independently incurs an additional cost burden of approximately 34%. This cost increment, independent of gestational age and major clinical confounders, reflects the increased complexity of managing SGA infants—including more intensive nutritional support, more frequent clinical monitoring, and longer transitional care.

This study has several limitations. First, the single-center retrospective design limits generalizability. Second, the SGA group comprised only 17 infants (6.3%), limiting statistical power for secondary outcomes and the precision of the regression coefficients. Third, long-term follow-up data were not available. Fourth, data on major neonatal complications such as necrotizing enterocolitis (NEC) and sepsis were not systematically collected in this retrospective dataset and therefore could not be adjusted for in the multivariable models. Unmeasured confounding from these clinical drivers of prolonged hospitalization cannot be excluded. Future prospective studies should incorporate these important clinical variables to provide more comprehensive risk adjustment. Fifth, although the GLM with Gamma distribution is robust for skewed outcomes, the small number of SGA infants warrants cautious interpretation of the effect estimates. Future larger-scale multicenter prospective studies are warranted to validate these findings.

## Conclusions

In this single-center cohort of very preterm infants, SGA was independently associated with approximately 40% longer LOS and 34% higher hospitalization costs after adjusting for gestational age, mode of delivery, IMV duration, and BPD. These findings highlight the need for targeted clinical management strategies and resource allocation for SGA very preterm infants.

## Data Availability

The datasets used and analyzed during the current study are available from the corresponding author on reasonable request.
